# Lescher on Milk-Sickness

**Published:** 1850-07

**Authors:** J. J. Lescher

**Affiliations:** Mt. Carmel, Ill.


### ARTICLE II.

*Milk Sickness.*—By J. J. Lescher, M. D. of Mt. Carmel, Ill.

In endeavoring to identify the poison of *milk-sickness* with Malaria, or, in other words, to argue the correctness of the belief, that the cause of Malarious fevers, and of *milk-sickness*, or *tires* in the brute are the same, I will not attempt an elaborate article, but rather present a brief statement of facts which would seem to give countenance to such a theory of the poison in question. To discover the pestiferous agent, has engaged the attention of the wise and the unwise; each one advancing a theory at war with all others. That there should be such discrepancies in opinion, has at all times been a matter of no little astonishment to me; inasmuch as so simple and plausible a solution of the mystery is at hand, and one which seems incontrovertible.

Whilst one class has evinced no little labor and research in ascribing the cause of *tires* to a vegetable origin, contending that the noxious weed had been discovered; another with an equal share of rhetorical skill, has as violently assailed it as preposterous, and instead of a vegetable, believed it a mineral.

A third, not content with either, confidently finds it in water, not knowing whence it derives, or by what name it is to be known. In arriving nearer the truth, as I conceive, however vaguely expressed, a fourth class, call it an exhalation from the surface of the earth, the chemical relation of which, they cannot define. That *milk sickness* is induced in the quadruped by an exposure to an earthy exhalation, and that Malaria is the exhalation, there can scarcely be a doubt, and for the following reasons:

Upon a careful inquiry concerning the location in which the disease under consideration prevails as an endemic, we invariably find that the same localities engender in man, malarious fevers proportioned in severity to the brute scourge. We find by reason of the prevalence of disease in man, the presence of Malaria; that the soil is alluvion, being a mixture of animal and vegetable remains. We find furthermore, that the periods most favorable for both classes of disease are identical.

Since we have every reason to believe that the disease in the brute spends its force on the nutritive system and abdominal viscera primarily, as is peculiarly manifested by visceral obstructions, a greatly depraved condition of the secretions, and a very marked derangement of the function of innervation; and knowing a set of conditions precisely similar, to manifest themselves in the human organism, following an exposure to miasm, should we not seem to approximate something like a just conception of cause and effect, in ascribing to the same pathological conditions a cause one and the same? To forestall the objections of those who would deny or reject my theory of the cause of *tires* on the ground that it is known to occur in places far remote from the vicinity of stagnant pools and marshes, so far indeed as to preclude the possibility of the Malaria having traveled thither, I would say: That in such places wheresoever they be, the soil, from its animal and vegetable composition, nevertheless gives Malaria, tho' in a degree less than in low and marshy places, and as a necessary consequence, we find ague, which of itself establishes beyond a doubt, the true character of the soil and miasm.

To the individual who will exclaim, "ah! but why does not milk-sickness show itself wherever ague, bilious fever, &c. do?" The answer may be easily discovered by inquiring into the mode and manner of raising cattle in such places. Instead of being permitted to run at large and endure the hardships of an exposure to the inclemencies of winter, and left to seek for a subsistence in the croppings of shrubs, etc., they are kept under shelter and generously fed, in which situation they remain during winter, until the season of vegetation makes its appearance, when they have health and vigor, sufficient to resist the pernicious effects of malaria, which may be evolved during the succeeding autumn. Whereas, on the other hand, such cattle as are left to themselves, in a state bordering on starvation, become so enfeebled as actually to invite the fury of that which excites the scourge—the miasmatic hydra.—The reason of the exemption of horses from the *tires*, is undoubtedly attributable to the same cause. Horses are seldom known to have it, and for the very simple reason, that they are rarely left in herds to graze the localities in which cows and other horned cattle are permitted to run. Sheep are exempt from the same cause.

Since no cause more terrible than that of malaria, can be discovered to account for these diseased phenomena; and from its known pernicious qualities with respect to the human organization, is it not more rational to adopt it as the cause which we so long have sought? It is surely more in accordance with the common sense of things, to adopt it, than to seek for and speculate on an unknown cause—an *ignis fatuus*.

Some may perhaps contend that the *milk-sickuess* in the brute is characterised by a greater virulence, than that of malarious diseases in man as occurring in the same localities. That such is the fact, I am not prepared to deny; but notwithstanding this seeming difficulty, I am not the less inclined to identify the cause. In the one case, by reason of constant exposure, the poison accumulates, and is likewise more highly concentrated, because of the uninterrupted inspiration of the same; and by reason of the peculiarity of its physical conformation, and the manner in which it must search for its food, the quadruped has its nostrils constantly brought in contact with the ground, in consequence of which, it inhales the denser and more poisonous part of the miasm. That such an explanation is strictly in accordance with facts is more than probable from the well known laws which govern malaria. No one who acknowledges such an agent as miasm, doubts that the lower strata of the poison are more noxious than those more elevated—that it cannot ascend to great heights, that the higher it ascends the more innocuous it becomes, until it ceases to be pernicious. And hence, the escape from diseases peculiarly traceable to a malarious origin, of the majority of those individuals who sleep in upper stories; of those whose houses are built upon an artificial or natural mound. If these laws are well established, and as tending to modify the virulence of the deleterious agent, taking it for granted that the same agent is the exciting cause of the *tires* in the brute, the difference in point of severity of the two diseases, can be very easily reconciled. For instance, upon two individuals of similar habits, expose to the same cause, differing only in degree, and the consequence will be, that, in the case of the one exposed to the lesser degree, he will be subjected to a disease precisely the same in character with the other, differing alone in severity. The same mode of reasoning with reference to the human and brute creatures, must lead to the same conclusions, modified to be sure, by the difference of organization.

If my etiology of *tires* be correct, and as such generally acknowledged by that portion of the profession whose habitation affords them the opportunity of witnessing and treating *milk-sckness*, as it shows itself in the human species, methinks very important and useful deductions, could be drawn, tending to a more rational, and eminently a more speedy and successful treatment. Instead of clothing it with gorgon terror and inexplicable mystery: Instead of regarding the disease under consideration, as induced by eating *poisoned* meat, and drinking *poisoned* milk, it were merely regarded as the consequence ot *diseased* ingesta, the treatment would be vastly simplified. and a long train of suffering, both mental and physical, would be spared the despairing sufferer. I italicise the word poisoned, because, when used in this connection, the mass imply by it something specifically alarming and terrible. Why not regard the diseased flesh and milk, as the flesh and milk of an animal dying of *brute ague*, as, indeed the vulgar name of *trembles*, which is but another appellation by which the *tires* is known, pathognomically expresses; meaning that it stands for a disease characterized by rigors.

The deleterious effects of diseased meats, on the human system is well known. Witness for instance, the history of those suffering under the effects of eating the flesh of cattle laboring under the disease which in Germany, is known as *milzbrand*, which, judging from the literal meaning of the term *milz*, spleen; and *brand*, gangrene, I am very much inclined to regard as synonymous with *trembles*. When the flesh of overdrawn oxen excites disease of the first passages in those eating it, the assertion of Prof. Dunglison to the contrary notwithstanding, an instance in proof of which, I will relate, it need not be a matter of surprise. But when the flesh of diseased cattle, and of over-driven cattle, excite symptoms very like each other, it should at least teach us caution in ascribing undue qualities to the flesh of *milk-sick* cattle.

A number of years past, whilst attending a course of lectures in the Medical College of Ohio, Prof. Mussey, related the following: During a hot and sultry autumnal day, a large and healty drove of fat oxen were for some cause or other overdriven, in reaching the Boston market, soon after which some were slaughtered, and the flesh disposed of. Many who had eaten of the meat, he observed, were seized with nausea, succeeded with violent pain in the stomach and bowels, incessant vomiting &c. Although at first, the bowels were frequently evacuated, they subsequently became obstinately constipated. An instance similar to the above, I remember to have seen in newspapers, a few years ago. Let us for a moment trace the disturbances in the system, following the use of the flesh of an ox which we have every reason to believe diseased preceeding slaughter. Sooner or later it excites a disordered condition of the stomach and bowels, known by the name of irritation of the gastro-enteric mucous lining membrane; which, if not speedily removed, excites through sympathy,the nerves of nutrition,and is soon succeeded by deranged function of the glandular system, causing the secretory organs to throw into the alimentary tube,irritating and depraved fluids, and finally terminating in inflamation of a low grade during the entire course of such diseased action, it may very properly be compared with *milk sickness.* To establish farther the identity of the cause of *tires* with malaria, we need but inquire into the means which are known to be effectual in destroying the cause. Cultivate the ground, and *milk-sickness*, disappears. The same holds good with respect to malaria, altho' not to such a degree as to prevent its formation totally. Prof. Drake, than whom we have not a sounder or more practical mind in the West, on the subject of Western Medical Topography, says, cultivation is the most infallible of all other means.

In the early settlement of Green Co., Ohio, the *trembles* was so annoying and of such frequent occurrence as very much to interfere with its settlement, which for a time remained at a stand-still, whilst neighboring counties not obnoxious to the scourge, rapidly filled up. However, as the lands were drained and cultivated, the disease disappeared, and at this time is perhaps wholly unknown to its citizens, except by name. In the same place, malarious fevers are by no means frequent.

Of the good effect of cultivation and draining, many incidents might be related; but let the experience of shrewd and observant farmers suffice. If asked how best they may protect their cattle, their answer is: "fence the infectious ground clear, and sow it in grass." Will not the same process diminish malaria? Is not malaria the noxious enemy against which the farmer directs his energies? Like the Apollo of heathen mythology, does he not stay the breath of the Python by destroying the monster? To return to a consideration of the different opinions concerning the cause of "brute ague." To detect the cause in any of the species of *rhus*, is so utterly groundless, as scarce deserving any thing more than a mere denial.

That it resides in any mineral, is equally as untenable; inasmuch as the means effectual in dissipating the cause, cannot reach the mineral.

That it must be malaria, is further confirmed by the fact, that wild turkeys contract the disease, which could scarcely be possible unless it be through the medium of their organs of respiration—unless, as some contend, they eat the diseased flesh.

In view of all the foregoing facts, if malaria be so subtle an agent, as to excite some of the most formidable diseases to be found in the calendar of human ills, where is the propriety of discarding it when searching for a thing known to possess properties alike with it? Or rather, wherein consists the impropriety of ascribing to it, powers capable of inducing functional and organic derangements in the brute creation, as it is known to effect in the human? There is no need of casting aside a known cause, to seek a bug-bear or a phantom.

Therefore, if malaria be the cause, we need no longer combat with a foe unshapen and unknown; but on the contrary give it "a local habitation and a name." The reproach of a baffled art would cease to rear its sneering front. But so long as there continues to be such a contrariety of opinions as to the etiology and pathology—so long as we seek for, and are confidently taught the presence of a *specific* poison in the fluids and solids of the brutes, imparting its specific qualities to the organism of the human being, engendering poisonous properties in the circulating mass, instead of regarding the fluids as deteriorated by reason of the irritation primarily excited and established in the first passages, so long may we deplore the want of a rational therapeutical management of the suffering—so long must we endure the taunts and jeers of the ignorant and petulant.

Upon the whole, undue importance seems to be attached to the disease. Prof. Drake says: "in the districts where the disease is endemic, it does not destroy as many as pleurisy or cholera morbus." From the crude notions of its pathology, its frequency is wildly exaggerated, confounded as it frequently is with Bilious Vomit, Gastritis, and intestinal derangement, produced by ordinary causes. And because such diseases are so often aggravated by an empiric course of stimulation and acrid purgation, under the impression that the disease under treatment is *Milk-Sickness*, lives are sacrificed, and the court of the insatiable *milk-sick* minister of Death is thronged with the ghosts of the unfortunate victims.

Milk-Sickness is generally regarded as an alarming disease, and as one demanding more than ordinary medication; the mass believing that the gift of curing it, is one akin to the supernatural, not to be acquired through the common media of medical instruction; and hence the propensity of ignorant and unprincipled medicasters to report simple gastric derangements as *Milk-Sickness*; the consequence being, that their fame reaps *milken* as well as *golden* laurels.

This lip-mocking class of trumpeters know, very well, the marvellousness of the mass, and the strange fondness which this same credulity begets for the mysterious, in the matter of disease and medicine. They know, likewise, the proneness of testing a physician's skill by a numerical method; that is, the one who loses the greater number of patients laboring under a *reputed* disease, however much skill he may have displayed, must bear the reproachful and *ungrateful* epithet of of an unsuccessful *doctor.* The palm of victory and triumph is awarded to the *pseudo-medicus.* The modest man of science, who treats *diseases*, must have his prized reputation tarnished and trodden under foot, whilst his rival, whom he does not condescend to regard as such, who treats *names*, changed to suit the popular breeze, is heralded as a prodigy in his profession. There is a strange disposition, in the great majority of men, to hanker after that which is not in the ordinary course of things. The every-day occurrences of life, move them not, but let something appear, which to them is incomprehensible, then all is wonder and amazement.

So it is in the practice of medicine. Simple and *truthful* names for diseases, as they ordinarily occur, excite no astonishment, unless it be a feeling akin to disappointment, because of the simplicity of the name of the disease. But bring forth the charlatan "Congstive Chills!" of "Winter Fever!" of "Milk-Sickness!" of "Fuss and Feath-ers," and witness the effect!—a whole community stands agog. The excitement whets the appetite of the panic-stirring *doctor;* as the season changes, so does his epidemic, always artful enough to take his cure from his superior, whose honest reputation he covets.

